# A randomized control trial of the effect of yoga on verbal aggressiveness in normal healthy volunteers

**DOI:** 10.4103/0973-6131.41034

**Published:** 2008

**Authors:** Sudheer Deshpande, H R Nagendra, Nagarathna Raghuram

**Affiliations:** Department of Yoga Research, Swami Vivekananda Yoga Anusandhana Samsthana, Bangalore, India

**Keywords:** Physical exercise, verbal aggression scale, Yoga

## Abstract

**Objective:**

To study the effect of yoga on verbal aggressiveness in normal healthy adults.

**Methods:**

Of the 1228 persons who attended introductory lectures, 226 subjects of both sexes who satisfied the inclusion and exclusion criteria and who consented to participate in the study were randomly allocated into two groups. These 226 subjects were between the ages of 17 and 62 years and 173/226 completed the eight weeks of intervention. The Yoga (Y) group practised an integrated yoga module that included asanas, pranayama, meditation, notional correction, and devotional sessions. The control group practised mild to moderate physical exercises (PE). Both groups had supervised practices (by trained experts) for one hour daily, six days a week for eight weeks.

Verbal Aggressiveness was assessed before and after eight weeks using the self-administered Verbal Aggressive Scale.

**Results:**

The baseline score of the two groups did not differ significantly (*P* = 0.66). There was a significant decrease in verbal aggressiveness in the yoga group (*P* = 0.01 paired samples t-test) with a nonsignificant increase in the PE group. ANCOVA using pre- values as covariates showed a significant difference between the groups (*P* = 0.013). RMANOVA for interaction between the sexes or age groups in change scores were not significant.

**Conclusions:**

This study has demonstrated that an eight week intervention of an integrated yoga module decreased verbal aggressiveness in the yoga group (in males and those below 25 years of age), with a nonsignificant increase in the PE group.

## INTRODUCTION

Although global scientific and technological progress is evidence of human intelligence and creativity, emotional hypersensitivity and aggression have increased.[1]

Violence remains one of the greatest public health threats to youth. Intentional injuries due to violence comprise the second leading cause of death of US adolescents,[2] as well as a substantial proportion of morbidity[3,4] such as elevated depressive symptoms and posttraumatic stress disorder.[5] Irritability and emotional outbursts are other manifestations of violence that could be measured. The verbal aggressiveness scale is a measure of violence that has been used in earlier studies.[6] Verbal aggressiveness is defined as an attack on an individual's self-concept instead of, or in addition to the person's position on a topic of communication, to inflict psychological pain.[7] A message must attack the self-concept of the receiver if it is to be considered as verbally aggressive message.[8] It was found that people who are high in the verbal aggression trait, differ significantly from those low in verbal aggression trait in terms of their use of these messages.[8]

Yoga which encompasses several techniques including physical postures, breathing techniques (Pranayama) and meditation has become very popular for its applications in health starting from better physical fitness[9] to a better quality of life in cancer patients.[10] Yoga has been used effectively for stress reduction that has resulted in biochemical[11] and physiological[12] changes. Several studies have highlighted the psychological benefits of integrated yoga practices such as anxiety, neurosis,[13,14] and depressive illness.[15,16] The clinical potential of yoga as a self-control technique for improving and stabilizing affective states was studied by Harvey. In a three armed study, Harvey compared yogic breathing exercises with two control groups (a course on the philosophy of meditation and a course in psychology) and demonstrated that yogic breathing exercises showed an improvement in mood and vigor as well as decreased tension, fatigue, and depression relative to subjects in control groups.[17] The mood benefits of Hatha yoga and swimming compared in college students showed that yoga was as effective as swimming in decreasing anxiety, confusion, tension and depression, and that the acute decreases after yoga were significantly greater than after swimming for men who were personally selected to participate.[18] Similar results have also been noted in psychiatric patients with a reduction in negative emotions factor in Profile of Mood States, including tension-anxiety, depression-dejection, anger-hostility, fatigue-inertia, and confusion-bewilderment after yoga.[19] The verbal aggressiveness scale was also used to assess the response of basketball players to the verbal aggressiveness of the coaches which showed that male players were more affected than the female players.[20]

Although there are several studies on the efficacy of yoga on different measures of emotional states, there are no studies on any measure of aggressive responses. Also there are no randomized control trials (RCTs) on the effect of yoga in comparison to PE in the same study. Hence, the aim of the current study was to investigate whether Yoga can provide benefits comparable to PE in reducing verbal aggressiveness in normal healthy adults.

## METHODS

### Subjects

Two hundred and twenty-six subjects who consented to participate in the study, were randomly allocated into two groups of equal size. The final data was available on 173 subjects. Inclusion criteria were (a) healthy individuals of both sexes and between the ages of 18 and 71 years, and (b) ability to read and write English because the participant had to fill up the questionnaire available in the English language. Exclusion criteria were (a) individuals with diseases such as diabetes, cancer, hypertension, anxiety, depression etc., (b) substance abuse, and (c) active nicotine abuse.

Source of subjects: Normal adult volunteers who consented to participate in the study were recruited from different locations in Bangalore.

Ethical clearance: Signed informed consent was obtained from all the subjects and also from the institutional heads where the classes were conducted. The institutional ethical committee of the parent institution had cleared the project proposal.

### Design

This was a prospective randomized control design to compare the efficacy of yoga (Y) with physical exercise (PE) as a control intervention in normal healthy volunteers. Motivational lectures were arranged in public centers such as colleges, health clubs, Rotary clubs, Lions' clubs and apartment complexes. The classes were planned in five different centers in the city of Bangalore.

After reading the instructions in the informed consent form about the design of the study, these subjects agreed to be in the allotted group. The experimental group was given Y practices and the control group was given PE for one hour daily on an empty stomach (6 to 7 a.m.). The classes were conducted six days a week for eight weeks and attendance was maintained by the teachers. Trained experts in either Y or PE conducted parallel sessions for the two groups in different rooms of the same building. It was ensured that there was no interaction between the subjects. The tests were administered on the first and last day of the study before starting the classes, by arranging the subjects to sit in a quiet hall, free from distractions and influences from each other, with supervisors moving around to clarify any doubts.

### Randomization

The subjects selected for the study were randomly allotted into two groups by using five different random number tables (different tables for each center) generated from the random number generator program.[21]

### Masking

The answered questionnaires were coded and kept away for future scoring. A psychologist who was not involved in the subject allocation or supervision of the classes, scored the questionnaires which were decoded only after the scoring of all answer sheets was completed.

### Assessments

The Verbal Aggressiveness Scale (VAS)—VAS [Table 1] is an interpersonal model and measure. The VAS developed by Infante and Wigley contains 20 items scored on a 5-point linear rating format with reverse scoring on ten out of 20 items (questions: 1, 3, 5, 8, 10, 12, 14, 15, 17, 20). The scores can range from 20 to 100. The VAS gives a single overall score that describes the disposition of an individual towards low, moderate, or high verbal aggressiveness. Scores from 20–46 suggest low verbal aggressiveness, 47–73 suggest moderate verbal aggressiveness and 74–100 suggest high verbal aggressiveness.

**Table 1:** VAS Questionnaire

1	I am extremely careful to avoid attacking individuals' intelligence when I attack their ideas.	1	2	3	4	5
2	When individuals are very stubborn, I use insults to soften their stubbornness.	1	2	3	4	5
3	I try very hard to avoid having other people feel bad about themselves when I try to influence them.	1	2	3	4	5
4	When people refuse to do a task I know is important without good reason, I tell them they are unreasonable.	1	2	3	4	5
5	When others do things that I regard as stupid, I try to be extremely gentle with them.	1	2	3	4	5
6	If individuals that I am trying to influence really deserve it, I attack their character.	1	2	3	4	5
7	When people behave in ways that are in very poor taste, I insult them in order to shock them into proper behavior.	1	2	3	4	5
8	I try to make people feel good about themselves, even when their ideas are stupid.	1	2	3	4	5
9	When people simply will not budge on a matter of importance, I lose my temper and say rather strong things to them.	1	2	3	4	5
10	When people criticize my shortcomings, I take it in good humor and do not try to get back at them.	1	2	3	4	5
11	When individuals insult me, I get a lot of pleasure out of really telling them off.	1	2	3	4	5
12	When I dislike individuals greatly, I try not to show it in what I say or how I say it.	1	2	3	4	5
13	I like poking fun at people who do things that are very stupid in order to stimulate their intelligence.	1	2	3	4	5
14	When I attach peoples' ideas, I try not to damage their self-concepts.	1	2	3	4	5
15	When I try to influence people, I make a great effort not to offend them.	1	2	3	4	5
16	When people do things that are mean or cruel, I attack their character in order to help correct their behaviour.	1	2	3	4	5
17	I refuse to participate in arguments when they involve personal attacks.	1	2	3	4	5
18	When nothing seems to work in trying to influence others, I yell and scream in order to get some movement from them.	1	2	3	4	5
19	When I am not able to refute others' positions, I try to make them feel defensive in order to weaken their positions.	1	2	3	4	5
20	When an argument shifts to personal attacks, I try very hard to change the subject.	1	2	3	4	5

1 – Almost never true, 2 – Rarely true, 3 – Occasionally true, 4 – Often true, 5 – Almost always true

Validity: This scale is stable across time. The reported test-retest reliability is 0.82 for a four week period. Further, cross-culture reliability has been supported in a number of studies.[7]

### Interventions

#### Yoga group

Table 2 shows the list of practices used for the two groups. The integrated yoga module was selected from the integrated set of yoga practices used in earlier studies on yoga for positive health.[22] The module was developed based on ancient Yoga texts[23] to bring about a total development at the physical, mental, emotional, social, and spiritual levels.[24] The techniques included i) physical practices (*Kriyas*, *asanas*, healthy yoga diet), ii) breathing practices with body movements and *Pranayama*, iii) meditation, iv) devotional sessions, v) lectures on yoga, vi) stress management based on yogic philosophy, and vii) lifestyle change through notional corrections for blissful awareness under all circumstances (action in relaxation). Qualified yoga teachers taught yoga.

**Table 2:** Details of Y and PE Practices

	Yoga practices		Physical exercise practice	
		
No.	Duration	Names	Duration	Names
1)	5 minutes	**Breathing practices**	10 minutes	**Warm up Exercises**
		Hands in and out breathing		(a) loosening of ankles
		Dog breathing		(b) knee caps
		Tiger breathing		(c) waist
		Straight leg raise breathing		(d) spine
				(e) twisting
2)	5 minutes	**Loosening Exercises**		(f) shoulder movements
		Jogging		(g) hands movement
		Forward and backward bending		(h) Wrist movements and rotations
		Side bending		(i) neck movement and rotations
		Twisting		(j) head movement and rotations
		Pavanamuktäsana kriya		
			5 minutes	**Stretches**
3)	25 minutes	**Äsanas**		(a) leg stretch
		**Standing**		(b) hand stretch
		Ardha Chakrasana		(c) leg to hand
		Pada Hastasana		(d) sideward leg stretch (full)
		Privritta Trikonasana		(e) folded leg lumber stretch
		**Sitting**		(f) dog stretch
		Vajrasana		(g) tiger stretch
		Supta Vajrasana		(h) dorsal stretch
		Chakrasana		
		Hamsasana or Mayurasana	10 minutes	Sit-ups (50 to 100 times)
		**Prone postures**		Push-ups (20 times)
		Dhanurasana		Squats
		**Supine postures**		
		Sarvangasana	10 minutes	**Supine**
		Matsyasana		(a) single leg raising
		Ardha Shirshasana or Shirshasana		(b) alternative leg raising
				(c) both leg raising (50 times)
				(d) coming up and touching the knees to forehead and going back
				(e) Cycling
4)	5 minutes	**Deep relaxation technique**	10 Minutes	**Supine rest** (Guided relaxation)
5)	10 minutes	**Pranayama**	10 minutes	**Dynamics**
		Kapalabhati		(a) forward Backward bending
		Vibhagiya Pranayama		(b) side bending
		Nadishuddhi Pranayama		(c) bending and twisting
		Sitali, Sitkari and Sadanta		(Simple and legs apart)
		Bhramari Pranayama		(d) Twisting
		Nada Anusandhana		
		Or	5 minutes	**Lectures**
6)		**Meditation—Om Meditation**		
7)	10 minutes	**Bhajans/Lectures**		

### Physical exercise group

The set of physical exercises chosen for this study consisted of standard practices[25] to provide mild to moderate exercises designed by experts in physical education and taught by trained physical education teachers. This group also had interactive lectures on healthy lifestyle including diet habits and stress management based on modern medical knowledge. The daily sessions began with short talks of five minutes on lifestyle and health covering the topics of (a) healthy diet (six talks) such as classification of foods, energy-yielding foods, role of animal fat and relation to cholesterol, vegetarian vs nonvegetarian diet, value of fiber etc., (b) value of exercise and health (six sessions) explaining different type of exercises, effects on muscles, joints, the value of regular sport activity etc, (c) bad effects of smoking (four talks), alcohol and other chemical abuse (two sessions), (d) effects of mental stress on health and the role of physical exercise in management of stress. This was followed by practice of the physical exercises for 45 minutes with enough rest in between. The sessions ended with ten minutes of self-relaxation (without guided instructions) in the supine position.

### Data extraction

The scoring of the questionnaires was carried out as per the instructions in the manual and under the guidance of a psychologist. They were decoded after the scoring of both pre- and post- data

### Data analysis

Data was analyzed using SPSS version 10.0. A sample size of 164 was calculated based on previous studies,[26] which showed an effect size of 0.8, with a power of 0.8 and alpha set to 0.05. This calculation was done using G power.[27] The size of the sample actually recruited was 226 while only data on 173/226 subjects were available for analysis.

The statistical tests used were paired samples t-test for pre-post comparison and ANCOVA for change score comparison of the two groups. Interaction between males and females in their change scores in yoga and control groups was checked by Repeated Measures ANOVA (RMANOVA). As the study population had a wide age range, analysis was also carried out by considering the median age of 25 years as the value for grouping them as juniors (age ≤ 25 years) and seniors (age > 25 years). The interaction between these two groups in their change scores were also checked by RMANOVA.

## RESULTS

**Figure 1:**
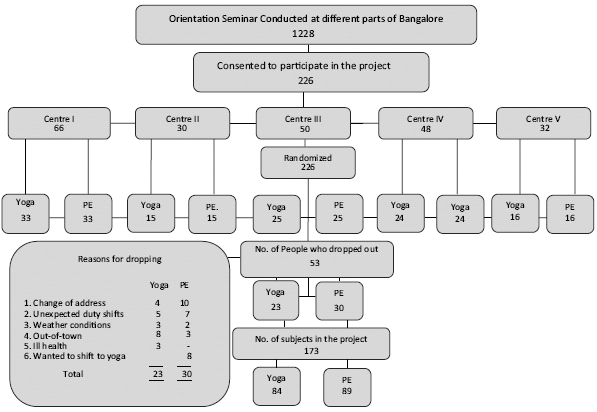
Trial profile

Figure 1 shows the trial profile of the 1228 subjects who attended the motivational lectures. Two hundred twenty-six subjects who satisfied the inclusion and exclusion criteria, were selected and randomly allotted to two groups: Y and PE. The reasons for drop-out of 53 subjects are shown in the figure. Data on 84 subjects in the yoga group and 89 in the control group were available for the final analysis.

Table 3 shows the demographic data. There were 80 females and 93 males within the age range of 17–62 years. The mean ages were 28.7 ± 11.6 years for the Y group and 30.8 ± 11.9 years for the PE group. They belonged to different categories such as college students, employees, business people and housewives. There were ten subjects with low scores, 156 subjects with moderate scores and seven subjects with high scores on the VAS.

**Table 3:** Demographic data

	Yoga (*n* = 84)	PE (*n* = 89)
Age (years)	28.73 ± 11.56	30.81 ± 11.86
Range (years)	17–67	18–62
Female	40	40
Male	44	49
Category of people		
Students	42	44
Employees	18	23
Housewives	14	12
Business	10	10

Table 4 shows the changes after the intervention in the two groups. The scores on VAS in the Y group (59.77 ± 7.51 to 57.36 ± 6.20) showed a significant decrease (*P* = 0.01). There was a nonsignificant increase in the PE group (58.71 ± 9.25 to 59.93 ± 8.63). There was a significant difference between the groups (*P* = 0.013) on ANCOVA considering the pre- values as covariates. RMANOVA for interaction between males and females (*P* = 0.68) and the two age groups (*P* > 0.50) showed no significant differences between groups.

**Table 4:** Results of VAS after the intervention in both groups

	Y				PE			
		
	*n*	Before	After	*P*^†^	*n*	Before	After	*P*^†^	P*
Whole group	84	59.77 ± 7.51	57.36 ± 6.20	0.017	89	58.71 ± 9.25	59.93 ± 8.63	0.268	0.013
Age ≤ 25years	47	60.31 ± 7.10	57.60 ± 6.32	0.072	41	58.31 ± 10.06	59.49 ± 8.83	0.532
Age > 25 years	37	59.15 ± 8.01	57.09 ± 6.14	0.126	48	59.02 ± 8.67	60.28 ± 8.54	0.346
Females	40	60.38 ± 7.96	57.74 ± 6.48	0.053	80	58.55 ± 8.97	61.25 ± 7.38	0.73
Males	44	59.23 ± 7.96	57.20 ± 6.48	0.156	49	58.84 ± 8.91	58.86 ± 7.38	0.987

Legend: *P*^†^ = significance pre-post within groups (paired † test) P* = significance between groups (ANCOVA with pre- values as covariates) *n* = Number Interactions between change scores (pre/post) between sexes (males/females) and the two age groups (≤ 25 / >25) in the yoga and control groups were checked by using RMANOVA that showed that there was no significant difference between the two groups (P > 0.5).

## DISCUSSION

This is a randomized control prospective study in normal adults comparing the effects of Yoga (Y) and physical exercise (PE) on verbal aggressiveness. This study has demonstrated that an eight weeks' intervention of an integrated yoga module decreased verbal aggressiveness in the yoga group with a nonsignificant increase in the PE group. RMANOVA for interactions of change scores showed no significant differences between the sexes and age groups in either the yoga or control groups.

A comparison of the baseline VAS scores used in another study by Wolf (used to validate the Rajas domain of another questionnaire called Vedic personality inventory) showed that the means of the baseline scores (59.23 ± 8.44) of our study group (*n* = 173) are comparable to their population (*n* = 240) in the USA (56.04 ± 17.08).[28]

The changes found after eight weeks of intervention although not very significant, suggest that continued practices may show greater degree of changes. The type of assessment tool used may also not be the most suitable one to bring out the subtle changes that may have occurred after the yoga practices.

A study on the relationship between verbal aggressiveness and state anxiety in sports by Alexandra *et al.*[20] showed that male basketball players were more affected by verbal aggressiveness of their coaches compared to female basketball players as assessed by VAS administered immediately after the game. In their study, they also observed a positive correlation between their anxiety and VAS scores in male players. It is known that yoga with its holistic approach uses several techniques to calm down the mind and reduce the anxiety state. Our earlier studies have shown that in community home girls and congenitally blind children, sympathetic tone reduced after yoga practices which resulted in significant decreases in resting heart rates and breath rates, thus reducing fear and anxiety.[29] The sympathetic tone reduction could be a valuable treatment modality for the reduction of anxiety. Another study on PT teachers also showed that yoga reduced their sympathetic activity after three months of yoga practices.[30] A significant reduction in anxiety scores was observed in patients with anxiety neurosis[31] after a yoga program. Based on these observations, we may suggest that the reduction in aggressiveness in the present study could be due to the reduction in their baseline anxiety and sympathetic reactivity.

The rate of violent victimization among 12 to 24 year-olds is nearly twice as high as that among adults ≥ 25 years (Bureau of Justice Statistics, 1996). In the present study, the changes observed in VAS after yoga practices suggest that yoga can be used for the reduction of violence.

According to the most widely used scriptural reference on yoga, the sage Patanjali[32] defines yoga as a technique for developing mastery over the modifications of the mind and goes on to highlight many techniques that help in achieving this mastery. They are classified under eight major streams including injunctions for social and personal behavior (yama niyama), body postures (asanas), breathing (pranayama), and meditation (pratyahara, dharana, dhyana, and samadhi) techniques that lead to mastery over any of the modifications in the mind. Furthermore, the sage Vasistha[24] in his famous work, Yoga Vasistha, defines yoga as a technique to slow or calm the mind directly through deep internal awareness. Hence, it was hypothesized that verbal aggressiveness, one of the manifestations of an uncontrolled fast mind, can be decreased by these techniques of yoga.

The strength of this study is the good sample size and the design in which the control group also had the same duration of interaction with the instructor and learnt nonyogic physical practices comparable to the integrated Yoga module. And the study population was taken from different parts of Bangalore from different socio-economic classes of the city.

Some limitations of the study were (a) this could not be a blinded RCT as yoga is a self-corrective learning process, (b) although we ensured that both groups had not done any yoga practices before recruitment, the possibility that the control group participants may have been exposed earlier to the concepts and philosophy of yoga (as it is widely available in Indian media) could not be ruled out, (c) although significant, the difference found after eight weeks of intervention was small, raising the utility of just an hour's practice in today's busy schedules. However, it may be possible that continued longer durations of practice may show greater degrees of changes. This was noticed in asthma and schizophrenia projects, where shorter yoga intervention did not result in any significant changes but greater significance was seen when the intervention was increased.[33] Furthermore, a justification for yoga intervention would be the potential for other health benefits with yoga (such as positive effects on blood pressure, well being etc) and the complications and costs associated with drug therapy as pharmaceutical intervention. Future studies are required to study the physiological indicators of anxiety that may correlate with VAS. Also, a third arm with only lectures for education may be included in future studies.

In summary, this randomized, prospective, single-blind, comparative study has shown the efficacy of Yoga in decreasing verbal aggressiveness. Hence, yoga may be recommended in schools to deal with the problem of violence among students, which is still a live issue in all parts of the world.
